# Prevalence and risk indicators of dental wear in a Saudi subpopulation

**DOI:** 10.3389/froh.2025.1702554

**Published:** 2025-12-08

**Authors:** Ahmed A. Madfa, Moazzy I. Almansour, Mohammad D. Aljanakh, Ahmed H. Albaqawi, Sami A. Almohefer, Saad M. Al-Zubaidi, Sarah S. Alajlan, Mohammed K. Alsaleh, Ghaida A. Alsulaiman, Faisal A. Alzabni, Abdulelah S. Alajlan

**Affiliations:** Department of Restorative Dental Science, College of Dentistry, University of Ha’il, Ha’il, Kingdom of Saudi Arabia

**Keywords:** epidemiology, eating behavior, smoking habits, social habits, Saudi subpopulation, dental wear

## Abstract

**Background:**

The objective of this study was to investigate the prevalence of dental wear (DW) and evaluate potential contributing factors in the Hail province of Saudi Arabia.

**Methods:**

A total of 386 individuals were involved in this cross-sectional study, which combined clinical assessments and questionnaire-based investigations. The clinical assessment using the Tooth Wear Index (TWI) was conducted. Each tooth was assigned a TWI score, and subsequently, individuals were classified into groups according to their level of risk. In addition to completing a demographic questionnaire, participants were also asked to respond to inquiries about their social, dietary, and dental health practices. The Chi-square test was used to investigate the association between the variables. The logistic regression analysis was also conducted to identify factors associated with tooth wear and its related clinical variables.

**Results:**

A comprehensive examination was conducted on all 386 patients, revealing that 219 individuals (56.73%) exhibited observable indications of DW. The study sample exhibited a prevalence of dentin exposure accompanied by sensitivity at a rate of 97 cases, representing 25.6% of the total sample. In the current investigation, a total of 111 individuals, representing 28.8% of the sample, were identified as requiring dental care as a result of DW. There was a significant association between the severity of DW and variables such as age, sex, and educational level (*p* < 0.05). A significant correlation was observed between diet, oral health practices, and tooth wear (*p* < 0.05). The analysis highlights diet, systemic health, brushing timing, region/type of tooth wear, symptoms, and treatment needs as significant predictors of tooth wear.

**Conclusions:**

The prevalence of DW in a Saudi subpopulation is relatively high. It is imperative that dentists provide guidance to their patients on oral hygiene habits and dietary selections that may contribute to significant tooth wear, as tooth wear is currently acknowledged as a substantial concern in the realm of oral health.

## Background

Dental wear (DW) refers to the enduring and irreversible loss of dental hard tissues, independent of the presence of bacterial plaque (1, 2). DW is a process that occurs partly due to physiological factors and is also influenced by age. However, the development of a pathological condition may occur when the teeth experience significant wear, leading to alterations in both their aesthetic and functional properties (3).

The clinical manifestation of DW is the result of a multifaceted interplay between biological, tribological, mechanical, and chemical factors, which collectively contribute to its permanent nature (4–9). Abrasion is the pathological loss of tooth structure that occurs due to mechanical interactions with external objects or materials, such as toothbrushes, dental instruments, or abrasive agents. Attrition refers to the mechanical wear resulting from tooth-to-tooth contact and is often associated with parafunctional activities, including bruxism. Erosion involves the chemical dissolution of dental hard tissues by acids, which may originate from intrinsic sources, such as gastric reflux, or extrinsic sources, such as dietary acids. Abfraction describes cervical lesions caused by biomechanical stress and flexural forces on teeth, leading to microfractures and material fatigue in the cervical region (10, 11).

Individuals with eating disorders or gastroesophageal reflux disease are at an increased risk of dental wear due to repeated exposure of the teeth to intrinsic acids, while extrinsic dietary acids, such as those from acidic foods and beverages, can further exacerbate tissue loss. The use of fruit juice, fruits, and carbonated beverages, together with their frequency and specific habits, could also be considered potential risk factors. The appearance of attrition is more prevalent in cases where there is bruxism. In the process of evaluating the probability of DW, additional behavioral factors must be taken into account. The extent of DW can be determined by the nature of the contact between the mandibular and maxillary teeth. Abrasion is commonly associated with the mechanical action of tooth brushing and the use of toothpastes, particularly in an already acidic environment (12).

An enormous epidemiological study has been dedicated to investigating the prevalence and underlying factors that contribute to DW in the adult population (4, 13–18). The prevalence of significant variations in numerous civilizations around the world was reported in the systematic review by Muts et al. (13). The prevalence of exposure to dentin resulting from DW was observed in 23.4% in Germany (14). In the Japanese population, a study found that 26.1% of the 1,088 people aged 15–89 years exhibited indications of erosive wear (15). Furthermore, a study conducted in Northern India examined DW among a sample of 965 male fertilizer workers. The participants, aged 19–58, were found to have a DW of 71.1% (16). Two risk factors associated with DW were age and brushing frequency, as indicated by a study conducted in Chengdu, China, that examined the prevalence of DW in adults (17). The Chilean study did not find significant differences in DW between men and women. The study revealed that DW was observed in 97.9% of the participants, with a higher prevalence in the anterior teeth. In addition, the study identified association between DW and age (18). The prevalence of DW in an adult population was found to be 80%, according to a study conducted in the Netherlands. Previous research has indicated that DW tends to be more severe among older people, particularly men compared to women, as well as those from lower socioeconomic backgrounds (4).

The data pertaining to Saudi Arabia lacks a comprehensive establishment. Based on the findings of Al-Zarea (19), a significant proportion of people residing in the northern region of Saudi Arabia exhibited DW, with almost 75% of the population affected. Utilization of dental abrasives, exposure to dust, frequency and brushing technique, as well as unilateral chewing, were identified as risk factors that contributed to DW. Al-Khalifa (20) reported that the prevalence of dentin exposure was 83.5%, while the prevalence of DW was 58.8%. The tooth wear rates in the eastern province of Saudi Arabia exhibited a discernible disparity based on gender and educational factors.

Despite growing global interest in DW, particularly its causes, patterns, and public health impact, research in Saudi Arabia remains relatively limited and fragmented. Existing studies from regions such as the Eastern Province and Northern areas have identified a high prevalence of DW, with several associated risk factors, including dietary habits, brushing technique, environmental exposures, and systemic health conditions. However, these findings are region-specific and may not accurately reflect the oral health status of populations in other parts of the country (19–21). Notably, the Hail province, located in northern Saudi Arabia, has not yet been the subject of any comprehensive epidemiological investigation into dental wear. This lack of region-specific data presents a significant knowledge gap, especially considering the possible variation in lifestyle, dietary patterns, oral hygiene habits, environmental conditions (e.g., desert dust), and access to dental care in Hail compared to other regions. Moreover, the absence of localized data limits the ability of dental professionals and public health policymakers to design targeted prevention and intervention programs tailored to the needs of the Hail population. Without a clear understanding of the prevalence, severity, and risk factors specific to this region, dental wear may remain underdiagnosed and improperly managed, potentially leading to reduced oral function, compromised aesthetics, and lower quality of life for affected individuals. Consequently, this study aims to fill this critical gap by investigating the prevalence and contributing factors of dental wear in the Hail province. The findings are expected to inform evidence-based strategies for prevention, early detection, and management of dental wear in this underserved population. Therefore, the objective of this study was to determine the prevalence of DW and assess potential contributing factors in the Hail province of Saudi Arabia. By using this knowledge, experts and public health professionals will be equipped to establish protocols and develop preventive strategies for the passive management of tooth wear in the Hail province.

## Method

### Study design and setting

This cross-sectional study was conducted at the Dental Polyclinics of the College of Dentistry, University of Hail, and several public dental clinics across Hail, Saudi Arabia. These clinics serve a diverse patient population from within the city and neighboring areas, making them suitable sites for data collection. The study took place in Hail Province, one of the major cities in the northern region of Saudi Arabia. All procedures were carried out in accordance with relevant ethical standards and regulations. Ethical approval was obtained from the Ethics Committee of the College of Dentistry, University of Hail (Approval No. H-2021-146). Informed consent was obtained from all participants prior to data collection.

### Study population

Participants in the study consisted of subjects who sought dental services at the Dental College of the University of Hail and several public dental clinics over the period extending from December 2021 to October 2023. The study sample consisted of people aged 20–60 who sought dental care at the College of Dentistry and a selection of public dental clinics chosen at random. The sample for this study consisted of persons who held Saudi nationality. The study included individuals aged between 20 and 60 years. Only participants with normal occlusal relationships, specifically classified as Class I, were considered. Subjects were required to provide informed consent and demonstrate willingness to comply with all study protocols. Additionally, only individuals assessed to be in good general health, without any clinical abnormalities or atypical medical histories, as determined by a general dental practitioner, were included in the study. Participants were excluded if they were using prosthetic appliances that covered the occlusal or incisal surfaces of their teeth. Individuals currently wearing orthodontic appliances were also excluded. Subjects with more than three missing teeth in either the maxilla or mandible were not eligible for inclusion. Teeth that were fractured, carious, retained roots, deciduous, or had traumatic lesions were excluded due to their increased susceptibility to damage. Third molars were also excluded from the analysis. Finally, individuals with systemic medical conditions or those undergoing pharmacological treatments for oral or dental illnesses were not considered for participation in the study.

### Sample size determination

The anticipated sample size was determined based on the study objectives, which aimed to determine the prevalence of DW among a cohort of adult volunteers from Saudi Arabia and to evaluate the extent of tooth wear that required restorative or prosthodontic treatment. The sample size was determined using the formula developed by Cochran for the determination of the sample size as follows (22):

$$N=\;\frac{Z\alpha \;\times P\;(1-P)}{D^{2}}$$

The variables in question are as follows: *N* represents the minimum sample size, *α* is set at 0.05 with a corresponding critical value of 1.96, Z*α* denotes the critical value of the Normal distribution at *α*/2 (for instance, in the case of a 95% confidence level), *P* represents the prevalence of the outcome of interest (specifically, dental wear among subjects, with a prevalence rate of 83.5% based on a recent study conducted in the Eastern Province of Saudi Arabia), and D represents the degree of precision. The suggested sample size consisted of 244 participants. The sample size was augmented in order to account for potential non-responses or incomplete responses. The ultimate sample size consisted of 386 participants.

### Interview

The questionnaires used in this study were developed based on a thorough review of the literature (9, 23) to ensure their relevance and comprehensiveness. Their validity was initially assessed through a pilot test with 30 practitioners and subsequently reviewed by faculty members from the Department of Restorative Dental Science at the College of Dentistry, University of Hail, in collaboration with subject matter experts. This process ensured that all items were appropriate, relevant, and clearly formulated. Following these validations, a structured questionnaire, available in both Arabic and English, was finalized for use in the study (Supplementary Material I).

A structured questionnaire was used to assess demographic, lifestyle, oral hygiene, and clinical factors associated with DW. Demographics included age, gender, socioeconomic status, education, and residence. Dietary habits recorded included consumption of carbonated beverages, energy drinks, fruit juices, acidic foods, and general diet quality. Systemic health was self-reported as medically fit or compromised, and smoking habits including, cigarette, pipe, or other tobacco use, were documented. Oral hygiene practices were evaluated by toothbrushing frequency, type of toothbrush, timing, and technique.

Prior to conducting interviews and dental examinations, verbal informed consent was obtained from all participants, who were informed of their right to withdraw from the study at any time without consequence. The purpose, objectives, and procedures of the study were clearly explained to each participant. All participants were required to be literate in Arabic or English, ensuring accurate comprehension and responses. To accommodate variations in language proficiency and enhance data accuracy, the questionnaire was administered through face-to-face interviews conducted by trained researchers. During these interviews, questions were clearly explained, and participants were encouraged to seek clarification as needed. This approach minimized potential misinterpretation of items and ensured inclusive, reliable, and standardized data collection across all participants. All interviews were conducted by trained researchers (SSA, MKA, FAA) undergoing structured calibration training to standardize the process. Although multiple interviewers were involved, the training and adherence to a standardized protocol ensured consistency and minimized variability across all interviews.

### Clinical examination

The standardized clinical dental examination techniques were used for all examinations, and the assessment of oral cavity health adhered to the principles set forth by the World Health Organization (WHO) (23). Examinations were performed under identical clinical conditions using a dental chair, overhead halogen light, compressed air, a mouth mirror, and a blunt probe following WHO guidelines. All examiners were calibrated and trained prior to data collection. The evaluation assessed wear on all teeth, excluding third molars, and classified lesions as abrasion (mechanical), attrition (tooth-to-tooth or parafunctional habits), erosion (chemical), abfraction (cervical lesions), or combination. Abrasion and abfraction were assessed and distinguished during clinical examination based on established criteria. Abrasion was identified as broad, shallow, or V-shaped cervical notches associated with mechanical factors such as improper toothbrushing or use of abrasive toothpaste. Abfraction was identified as wedge-shaped cervical defects resulting from biomechanical stress and flexural forces, typically occurring at sites not subjected to direct mechanical contact. Examiners were trained and calibrated, and differentiation considered lesion morphology, location, and patient history of mechanical habits or parafunctional activity. The examination of the temporomandibular joint (TMJ) and masticatory muscles was performed after a scoring procedure (9, 24). Clinical examination included assessment of the TMJ for normal function or noise, and evaluation of the masseter and temporalis muscles for pain, tenderness, or enlargement. Ultimately, individual requirements for prosthetic and restorative care were determined for each patient. The need for prosthetic or restorative treatment was determined based on the Tooth Wear Index (TWI) scores and the clinical impact on function and aesthetics. Teeth with moderate to severe wear (TWI ≥ 2–3) exhibiting dentin exposure, reduced occlusal vertical dimension, sensitivity, or functional impairment were considered for restorative intervention. The decision also incorporated patient-reported symptoms and the potential for further wear or structural compromise.

### Grading of tooth wear

The scoring criteria to evaluate the incisal and occlusal surfaces of all teeth, as outlined in the Tooth Wear Index (TWI) devised by Smith and Knight (25), are presented in Table 1. Dental surfaces were assigned scores ranging from 0 to 4 based on the degree of tooth wear observed.

**Table 1:** Ordinal scale used for grading severity of tooth wear.

Grade	Surface	Degree of tooth wear
0	B/L/O/I	No loss of enamel surface characteristics
1	B/L/O/I	Loss of enamel surface characteristic
2	B/L/O	Loss of enamel, exposing dentine on less than one-third of surface
3	I	Loss of enamel, just exposing dentin
	B/L/O	Loss of enamel, exposing dentin on more than one third of surface
4	I	Loss of enamel and substantial loss of dentin
	B/L/O	Complete enamel loss, pulp exposure, secondary dentin exposure
	I	Pulp exposure or exposure of secondary dentin

Scoring according to (TWI) (B, buccal; L, lingual; O, occlusal; I, incisal).

Before commencing the study, the examiners (SSA, MKA, FAA) underwent a structured calibration training program to ensure accuracy, consistency, and standardization of clinical assessments. The training was supervised by senior clinicians (AAM, MIA, MDA) with over 10 years of experience in dental evaluation. These experts provided continuous guidance throughout the study and actively participated in the assessment of dental wear. In cases of diagnostic discrepancies, examiners engaged in collaborative discussions to reach consensus, thereby enhancing reliability. To further evaluate the consistency of the clinical assessments, a randomly selected subgroup of 30 patients underwent a second examination by the same examiner after a two-week interval. This interval minimized recall bias while maintaining clinical stability. Intra-examiner agreement was assessed using Cohen's kappa statistic, with both simple (unweighted) and weighted kappa values calculated, providing a robust measure of the reproducibility and reliability of the clinical observations.

### Statistical analysis

Data analysis was performed using the Statistical Package for the Social Sciences (SPSS) version 21 (IBM Corp., Chicago, IL, USA). Descriptive statistics summarized the demographic and clinical characteristics of the study population. DW was considered the dependent variable, while independent variables included demographics, diet, systemic health, oral hygiene, clinical assessments, tooth wear characteristics, and prosthetic/restorative needs. All variables were appropriately coded with reference categories to evaluate their independent associations. The Chi-square test was used to examine associations between DW and potential contributing factors. Addtionally, logistic regression models were developed to identify variables significantly associated with DW and related clinical parameters. A *p*-value of <0.05 was considered statistically significant. Based on clinical findings, necessary restorative interventions were also identified and documented.

## Results

### Reliability of clinical assessments

Intra-examiner agreement for the evaluation of tooth wear was high, with Cohen's Kappa values of 0.81 (simple) and 0.86 (weighted), indicating excellent reproducibility. Table 2 summarizes the socio-demographic characteristics, systemic health status, and behavioral factors of the 386 participants included in the study.

**Table 2:** Socio-demographic characteristics, systemic health, dietary habits, and oral hygiene practices of the study population (*n* = 386).

Variable		N (%)
Socio-demographic variables		
Age group (Year)	20–30	127 (32.9)
	31–40	111 (28.8)
	41–50	89 (23.1)
	Above 50	59 (15.3)
Gender	Female	206 (53.4)
	Male	180 (46.6)
Socioeconomic status	Low (6,000 and below)	237 (61.4)
	Moderate (6,000–12,000)	122 (31.6)
	High (Above 12,000)	27 (7)
Education	None	57 (14.8)
	Primary	73 (18.9)
	Secondary	136 (35.2)
	Bachelor or above	120 (31.1)
*Systemic condition, dietary habits, and social habits*		
Diet	Beverages (Carbonated “Cola”-Energy Drink-Fruit Juice)	161 (41.7)
	Acidic Food (Orange, Grapefruit, Lemon, Ketchup, Citrus Fruit)	131 (33.9)
	Healthy diet	94 (24.4)
Systemic condition	Medically fit	299 (77.5)
	Medically compromised	87 (22.5)
Smoking	Yes	76 (19.7)
	No	310 (80.3)
Pipe smoking	Yes	2 (0.5)
	No	384 (99.5)
Cigarette smoking	Yes	73 (19)
	No	312 (81)
Tobacco using (shama)	Yes	2 (0.5)
	No	384 (99.5)
Tooth brushing	Yes	244 (63.2)
	No	142 (36.8)
Type of toothbrush	No use	141 (36.5)
	Soft	112 (29)
	Medium	118 (30.6)
	Hard	15 (3.9)
Brushing time	No use	141 (36.5)
	Before meal	92 (23.8)
	After meal	67 (17.4)
	Both	86 (22.3)
Brushing technique	No use	141 (36.5)
	Correct	161 (41.7)
	Incorrect	84 (21.8)
Total (n)		386 (100)

### Prevalence and distribution of dental wear

Table 3 summarizes the clinical examinations, patterns of tooth wear, and restorative treatment needs among the study participants. TMJ assessment showed that the majority of participants had normal findings (88.6%), while clicking and noise were observed in 11.1% and 0.3%, respectively. Examination of the masticatory muscles revealed normal findings in 92% of participants, with tenderness in 5.2%, enlargement in 2.3%, and pain in 0.5%. Regarding tooth wear, 43.3% of participants showed no wear. Wear was most commonly observed in the mandibular anterior region (12.7%) or as a combination of two surfaces (28.2%). Occlusal surfaces were the most affected site (36%), followed by lingual (4.1%) and cervical (1%) surfaces. Sensitivity was reported by 25.6% of participants, whereas 2.4% experienced pain. Attrition due to parafunctional habits was the predominant type of wear (35.8%), followed by erosion (4.4%), abrasion (1.8%), abfraction (0.3%), and combination lesions (14.5%). The tooth wear index (TWI) scores indicated that 44.6% had a score of 0, 22.5% scored 1, 25.1% scored 2, and 7.8% scored 3. Restorative treatment was required in 28.8% of participants, while 71.2% did not need any restorative intervention.

**Table 3:** Clinical examination findings, tooth wear distribution, and restorative treatment needs of the study population (*n* = 386).

Basic characteristics	Assessment parameters	*N* (%)
TMJ examination	Normal	342 (88.6)
	Clicking	43 (11.1)
	Noise	1 (0.3)
Masticatory muscle exam (Masseter, Temporalis)	Normal	355 (92)
	Pain	2 (0.5)
	Tenderness	20 (5.2)
	Enlargement	9 (2.3)
Region of tooth wear	No wear	167 (43.3)
	Maxillary Anterior	29 (7.5)
	Maxillary Posterior	2 (0.5)
	Mandibular Anterior	49 (12.7)
	Mandibular Posterior	2 (0.5)
	Combination of 2 surfaces	109 (28.2)
	Combination of 3 surfaces	7 (1.8)
	Combination of all surfaces	21 (5.1)
Site of tooth wear	No wear	167 (43.3)
	Occlusal	139 (36)
	Lingual	16 (4.1)
	Cervical	4 (1)
	Combination of 2 surfaces	51 (13.2)
	Combination of 3 surfaces	5 (1.3)
	Combination of all surfaces	4 (1)
Symptoms	No symptoms	272 (72)
	Pain	9 (2.4)
	Sensitivity	97 (25.6)
Type of tooth wear	No wear	167 (43.3)
	Abrasion (Mechanical)	7 (1.8)
	Attrition (parafunctional habit, tooth-to-tooth)	138 (35.8)
	Erosion (chemical)	17 (4.4)
	Abfraction (cervical area)	1 (0.3)
	Combination	56 (14.5)
Tooth wear index	0	172 (44.6)
	1	87 (22.5)
	2	97 (25.1)
	3	30 (7.8)
Restorative treatment need	Yes	111 (28.8)
	No	275 (71.2)

### Associations of TWI with various demographic, behavioral, and clinical factors

Table 4 presents the association between the TWI and various demographic, behavioral, and clinical factors. The findings revealed significant associations between DW severity and age, sex, and educational level (*p* < 0.05). Older participants and males exhibited higher rates of tooth wear, while lower educational attainment was linked to increased severity. Socioeconomic status was not significantly associated with DW (*p* > 0.05). Consumption of acidic foods and beverages, systemic health status, smoking, and exposure to second-hand smoke were significantly correlated with DW (*p* < 0.05). No significant association was observed with pipe smoking or other forms of tobacco use (*p* > 0.05). Oral hygiene factors including brushing frequency, type of toothbrush, replacement intervals, and brushing technique were significantly related to DW (*p* < 0.05). Examination of the TMJ and masticatory muscles also showed significant associations with DW severity (*p* < 0.05).

**Table 4:** Distribution of tooth wear index across demographic, behavioral, and clinical variables in the study population (*n* = 386).

Variables		Tooth wear index				*P* value
		0	1	2	3	
Age group	20–30 years	67 (52.8%)	34 (26.8%)	23 (18.1%)	3 (2.4%)	<0.001
	31–40 years	59 (53.2%)	21 (18.9%)	29 (26.1%)	2 (1.8%)	
	41–50 years	34 (38.2%)	20 (22.5%)	25 (28.1%)	10 (11.2%)	
	Above 50 years	12 (20.3%)	12 (20.3%)	20 (33.9%)	15 (25.4%)	
Gender	Female	146 (70.9%)	38 (18.4%)	18 (8.7%)	4 (1.9%)	<0.001
	Male	26 (14.4%)	49 (27.2%)	79 (43.9%)	26 (14.4%)	
Socioeconomic status	High	13 (48.1%)	11 (40.7%)	2 (7.4%)	1 (3.7%)	0.191
	Moderate	53 (43.4%)	27 (22.1%)	32 (26.2%)	10 (8.2%)	
	Low	106 (44.7%)	49 (20.7%)	63 (26.6%)	19 (8.0%)	
Education	None	11 (19.3%)	9 (15.8%)	22 (38.6%)	15 (26.3%)	<0.001
	Primary school	29 (39.7%)	17 (23.3%)	22 (30.1%)	5 (6.8%)	
	Secondary school	81 (59.6%)	27 (19.9%)	24 (17.6%)	4 (2.9%)	
	Bachelor or above	51 (42.5%)	34 (28.3%)	29 (24.2%)	6 (5.0%)	
Diet	Beverages	92 (57.1%)	32 (19.9%)	30 (18.6%)	7 (4.3%)	<0.001
	Acidic Food	58 (44.3%)	34 (26.0%)	28 (21.4%)	11 (8.4%)	
	Healthy diet	22 (23.4%)	21 (22.3%)	39 (41.5%)	12 (12.8%)	
Systemic condition	Medically fit	146 (48.8%)	70 (23.4%)	68 (22.7%)	15 (5.0%)	<0.001
	Medically compromised	26 (29.9%)	17 (19.5%)	29 (33.3%)	15 (17.2%)	
Smoking	Yes	53 (69.7%)	15 (19.7%)	6 (7.9%)	2 (2.6%)	<0.001
	No	119 (38.4%)	72 (23.2%)	91 (29.4%)	28 (9.0%)	
Pipe smoke	No	170 (44.3%)	87 (22.7%)	97 (25.3%)	30 (7.8%)	0.475
	Yes	2 (100.0%	0 (0.0%)	0 (0.0%)	0 (0.0%)	
Cigarette smoke	No	121 (38.8%)	72 (23.1%)	91 (29.2%)	28 (9.0%)	<0.001
	Yes	50 (68.5%)	15 (20.5%)	6 (8.2%)	2 (2.7%)	
Tobacco	No	170 (44.3%)	87 (22.7%)	97 (25.3%)	30 (7.8%)	0.475
	Yes	2 (100.0%	0 (0.0%)	0 (0.0%)	0 (0.0%)	
Tooth brushing	Yes	73 (29.9%)	72 (29.5%)	81 (33.2%)	18 (7.4%)	<0.001
	No	99 (69.7%)	15 (10.6%)	16 (11.3%)	12 (8.5%)	
Type of toothbrush	No use	98 (69.5%)	15 (10.6%)	16 (11.3%)	12 (8.5%)	<0.001
	Soft	33 (29.5%)	37 (33.0%)	34 (30.4%)	8 (7.1%)	
	Medium	38 (32.2%)	32 (27.1%)	40 (33.9%)	8 (6.8%)	
	Hard	3 (20.0%)	3 (20.0%)	7 (46.7%)	2 (13.3%)	
Brushing time	No use	98 (69.5%)	15 (10.6%)	16 (11.3%)	12 (8.5%)	<0.001
	Before meal	19 (20.7%)	29 (31.5%)	34 (37.0%)	10 (10.9%)	
	After meal	23 (34.3%)	24 (35.8%)	18 (26.9%)	2 (3.0%)	
	Both (before & after)	32 (37.2%)	19 (22.1%)	29 (33.7%)	6 (7.0%)	
Brushing technique	No use	98 (69.5%)	15 (10.6%)	16 (11.3%)	12 (8.5%)	<0.001
	Correct	43 (27.4%)	50 (31.8%)	52 (33.1%)	12 (7.6%)	
	Incorrect	29 (35.8%)	20 (24.7%)	26 (32.1%)	6 (7.4%)	
TMJ examination	Normal	168 (49.1%)	74 (21.6%)	79 (23.1%)	21 (6.1%)	<0.001
	Clicking	4 (9.3%)	12 (27.9%)	18 (41.9%)	9 (20.9%)	
	Noise	0 (0.0%)	1 (100.0%)	0 (0.0%)	0 (0.0%)	
Masticatory muscle exam	Normal	171 (48.2%)	79 (22.3%)	81 (22.8%)	24 (6.8%)	<0.001
	Pain	0 (0.0%)	0 (0.0%)	1 (50.0%)	1 (50.0%)	
	Tenderness	1 (5.0%)	5 (25.0%)	10 (50.0%)	4 (20.0%)	
	Enlargement	0 (0.0%)	3 (33.3%)	5 (55.6%)	1 (11.1%)	

### Multivariate analysis of factors associated with DW

Logistic regression analysis identified several factors associated with tooth wear in the study population (Table 5). Consumption of acidic foods (OR = 0.40, 95% CI: 0.06–0.81, *p* = 0.036) and being medically fit (OR = 0.38, 95% CI: 0.15–0.83, *p* = 0.02) were associated with lower odds of tooth wear, while brushing before meals increased the odds (OR = 2.65, 95% CI: 1.08–6.57, *p* = 0.034). Wear in the mandibular anterior teeth (OR = 0.07, 95% CI: 0.02–0.31, *p* < 0.001) and involvement of two surfaces (OR = 0.14, 95% CI: 0.04–0.43, *p* = 0.001) were significant compared to wear across all surfaces. Participants reporting pain had higher odds of tooth wear (OR = 7.18, 95% CI: 1.20–43.2, *p* = 0.031), and abrasion as a single type of wear was associated with lower odds compared to combined wear types (OR = 0.037, 95% CI: 0.005–0.57, *p* = 0.018). The need for prosthetic or restorative treatment was strongly associated with tooth wear (OR = 56.9, 95% CI: 30.7–107.8, *p* < 0.001). Other variables, including age, sex, socioeconomic status, education, beverage intake, smoking, brushing technique, and TMJ or masticatory muscle findings, were not significantly associated with tooth wear.

**Table 5:** Logistic regression analysis of factors associated with tooth wear and related clinical variables in the study population (*n* = 386).

Variables		Odds ratio	95% CI (lower–upper)	*P*-value
Age group	20–30 years	0.65	0.21–2.04	0.464
	31–40 years	1.19	0.39–3.60	0.757
	41–50 years	0.97	0.33–2.88	0.957
	Above 50 years	Referent	–	–
Sex	Male	0.42	0.06–2.77	0.369
	Female	Referent	–	–
Socioeconomic status	High	0.58	0.07–4.99	0.617
	Moderate	0.91	0.40–2.08	0.824
	Low	Referent	–	–
Education	None	0.86	0.28–2.63	0.787
	Primary school	0.46	0.15–1.38	0.165
	Secondary school	0.59	0.22–1.59	0.300
	Bachelor or above	Referent	–	–
Diet	Beverages	0.59	0.25–1.40	0.228
	Acidic food	0.40	0.06–0.81	0.036
	Healthy diet	Referent	–	–
Systemic condition	Medically fit	0.38	0.15–0.83	0.020
	Medically compromised	Referent	–	–
Smoking	Yes	1.20	∼0—extremely large	1.000
	No	Referent	–	–
Tooth brushing	Yes	0.13	∼0—extremely large	0.999
	No	Referent	–	–
Type of toothbrush	No use	0.79	∼0—extremely large	1.000
	Soft	1.00	0.22–4.59	0.996
	Medium	0.68	0.15–3.11	0.618
	Hard	Referent	—	—
Brushing time	No use	Referent	—	—
	Before meal	2.65	1.08–6.55	0.034
	After meal	2.27	0.74–6.92	0.149
	Both	Referent	—	—
Brushing technique	Correct	1.52	0.36–3.54	0.328
	Incorrect	Referent	—	—
TMJ examination	Normal	5.59	∼0—very large	0.665
	Clicking	7.33	∼0—very large	0.618
	Noise	Referent	–	–
Masticatory muscle exam	Normal	0.61	0.12–3.13	0.551
	Pain	1.29	0.04–39.89	0.885
	Tenderness	3.16	0.46–21.45	0.240
	Enlargement	Referent	—	—
Region of tooth wear	No wear	∼0	∼0—extremely large	0.858
	Max. Ant.	0.39	0.09–1.66	0.202
	Max. Post.	0.29	0.00–37.75	0.621
	Mand. Ant.	0.07	0.02–0.31	0.000
	Mand. Post.	0.65	0.02–21.24	0.806
	Combination of 2 surfaces	0.14	0.04–0.43	0.001
	Combination of 3 surfaces	0.14	0.01–1.42	0.096
	Combination of all	Referent	—	—
Site of tooth wear	No wear	Referent	—	—
	Occlusal	0.35	0.03–4.43	0.421
	Lingual	0.07	0.00–2.38	0.142
	Cervical	9.31	0.13–656.68	0.304
	Combination of 2 surfaces	0.38	0.03–4.35	0.440
	Combination of 3 surfaces	0.59	0.03–14.13	0.745
	Combination of all surfaces	Referent	—	—
Symptoms	Pain	7.18	1.20–43.2	0.031
	Sensitivity	1.79	0.88–3.66	0.109
	No symptoms	Referent	—	—
Type of tooth wear	Abrasion	0.037	0.005–0.57	0.018
	Attrition	0.65	0.24–1.73	0.388
	Erosion	0.54	0.05–6.11	0.619
	Abfraction	0.68	0.00–92.90	0.877
	Combination of 2 types	Referent	—	—
Prosthetic/Restorative treatment need	Yes	56.9	30.7–107.8	0.000
	No	Referent	—	—

## Discussion

The findings of this study revealed significant associations between DW and variables such as age, sex, education, diet, oral hygiene, smoking, and systemic health, suggesting that these factors play a meaningful role in the prevalence and severity of DW in this population.

The present study identified DW in 56.73% of participants. Previous studies report DW prevalence ranging from 57% to 85% (4, 18, 23, 26–28). The prevalence observed here was higher than rates reported in Germany (14) and Ireland (18), placing it at the upper end of global estimates. The higher prevalence of dental wear in Hail compared to Germany and Ireland is likely due to a combination of factors, including more frequent consumption of acidic foods and beverages, improper brushing habits, parafunctional behaviors like bruxism, and differences in preventive dental care. Methodological variations between studies may also contribute to the observed differences. These findings highlight the need for targeted dental care in this age group, as tooth wear increases with age.

DW was more prevalent in incisors than in other teeth, regardless of mandibular or maxillary location, a pattern consistent with previous studies (29–31). Factors contributing to higher incisor wear include thinner enamel, greater functional involvement, longer retention in older adults, and prolonged exposure due to early eruption (29).

Age emerged as the most significant factor associated with DW across all tooth categories. Evidence suggests that DW is already observable in adolescents (23, 32), and in this study, notable tooth wear was detected beginning in adolescence. Physiological wear accumulates with aging, especially among older adults, affecting tooth function, structure, and aesthetics (3), consistent with prior research (23, 33).

Sex differences in DW were also observed, with males exhibiting higher prevalence than females, in line with previous studies (14, 20). Increased masticatory force, stress, dietary habits, and delayed dental care in males likely contribute to this disparity (8, 20, 34–36). Notably, cigarette smoking was significantly associated with greater DW, suggesting that smoking may partly account for the observed sex differences in tooth wear.

Education level was significantly correlated with DW. Individuals with higher education tend to maintain better oral hygiene and healthier diets, which may mitigate tooth wear (20, 37, 38). However, some studies, such as one conducted in the UK, reported a positive correlation between higher education and DW, reflecting differences in dental perceptions and acceptance (39). This seemingly paradoxical finding may be explained by differences in lifestyle, dietary preferences, and cultural perceptions of oral health. For example, individuals with higher education may consume more acidic beverages such as wine, fruit juices, or carbonated drinks, or they may engage in behaviors such as frequent toothbrushing with abrasive toothpastes, which can increase wear. In addition, variations in dental awareness and perceptions of what constitutes acceptable levels of wear could influence both reporting and diagnosis. These findings highlight the complex interplay between education, behavior, and cultural context in shaping the risk of dental wear (30–32).

Our analysis revealed a significant association between lower educational attainment and increased dental wear, which may be attributed to limited oral health literacy and reduced access to preventive dental care. Studies have shown that individuals with lower education levels often exhibit poorer oral health behaviors and higher rates of dental issues, including enamel demineralization. Additionally, lower educational attainment is linked to decreased utilization of preventive dental services, further contributing to oral health disparities (40, 41).

Although no statistically significant association was found between DW and socioeconomic status, participants in the lowest socioeconomic category demonstrated higher DW prevalence, consistent with previous reports (27). This relationship may be influenced by dietary habits, lifestyle, oral health knowledge, and access to dental care.

Acidic food and beverage consumption was positively associated with dental wear (42–44), supporting evidence that frequent exposure to low-pH substances, such as soft drinks and citrus fruits, promotes enamel and dentin demineralization and increases susceptibility to mechanical abrasion.

Oral hygiene practices, including brushing frequency and toothbrush type, were significantly related to DW (45). Mechanical friction during tooth brushing accelerates wear, particularly when medium bristle brushes are used or when toothbrushes are replaced frequently (46, 47). The present study indicated that tooth wear is influenced more by brushing habits. Participants who brushed regularly showed greater mechanical abrasion, especially when using hard or medium bristles, brushing before meals, or applying incorrect technique. In contrast, those who brushed with soft bristles, used proper technique, and brushed after meals exhibited significantly less wear. These findings suggest that patient education should emphasize gentle brushing with soft bristles, correct technique, and appropriate timing to minimize enamel loss while maintaining oral hygiene (48).

Smoking was associated with higher DW prevalence, consistent with previous findings (9, 48, 49). Smoking-related lesions may result from local frictional and vascular effects (50). In this study, cigarette smoking was significantly associated with dental wear, whereas pipe and other tobacco use were not. This discrepancy is most plausibly explained by differences in exposure intensity and sample size. Only two participants reported pipe or smokeless-tobacco use, providing inadequate statistical power to detect a true effect and increasing the likelihood of a type II error (51). Cigarette use involves greater cumulative exposure to heat, chemicals, and altered salivary pH, and is linked to parafunctional habits like bruxism, all contributing to enamel wear (52). The nonsignificant findings for pipe or smokeless tobacco likely reflect sample size rather than a true lack of effect.

Finally, a significant association was found between masticatory muscle pain and tooth deterioration (24). Persistent muscle activation and parafunctional habits, such as clenching, increase forces on the teeth, potentially leading to muscle discomfort, temporomandibular joint disorders, and structural damage to teeth and supporting tissues (24).

## Clinical implications

DW is highly prevalent in Hail province and poses significant functional and aesthetic challenges. Clinicians should prioritize routine screening for DW, particularly in patients with risk factors such as older age, male sex, lower educational attainment, medically compromised status, frequent acidic food consumption, and improper brushing habits. Preventive strategies should include counseling on gentle brushing with soft bristles, avoiding brushing immediately after acidic meals, and moderating intake of acidic foods and beverages. For patients already exhibiting tooth wear, early restorative intervention—such as minimally invasive composite restorations or protective occlusal splints—can prevent progression and preserve tooth structure (53–55). Additionally, public health initiatives and clinician training should emphasize risk assessment, patient education, and tailored preventive care, ensuring effective management of DW within the local dental care system.

This study is the first to examine the prevalence and contributing factors of DW among adults in Hail province, providing valuable data to support local oral health planning and targeted preventive strategies. However, caution is warranted when generalizing these findings to other populations due to demographic differences and variations in study methodologies. Limitations include the cross-sectional, incomplete assessment of potential risk factors—such as parafunctional habits and lifestyle—and challenges in differentiating mechanical from chemical causes of DW during clinical evaluation. The use of the TWI rather than more recent indices such as the Tooth Wear Evaluation System (TWES) (56), which provide more detailed criteria to distinguish between attrition, erosion, and abrasion/NCCLs. While TWI is widely validated and suitable for epidemiological studies, it may not capture the subtle differences between the specific types of wear as precisely as newer indices. Future studies could consider using TWES or similar systems to allow more detailed characterization of tooth wear patterns. Future research should adopt longitudinal designs with larger and more representative samples to clarify the temporal relationship between risk factors and DW progression. Comprehensive evaluation of additional behavioral, dietary, and systemic determinants—including parafunctional habits, lifestyle factors, and occupational exposures—would enhance understanding of DW etiology.

## Conclusions

Within the limitations of this cross-sectional investigation, the present study revealed a notable prevalence of DW among adults in the Hail Province of Saudi Arabia. The occurrence and severity of DW were influenced by multiple factors, including dietary habits, oral hygiene practices, systemic health, and socioeconomic and demographic variables. Older age, male gender, lower educational attainment, and less favorable socioeconomic conditions were particularly associated with increased risk.

Understanding the multifactorial nature of DW is essential for preventing lesion progression and the development of new defects. Targeted preventive strategies should focus on modifying risk behaviors such as frequent consumption of acidic foods and beverages, improper brushing techniques, and smoking. Dentists are encouraged to incorporate comprehensive risk assessments—including evaluation of diet, biological factors, and systemic health—into routine care. Three-tiered prevention measures emphasizing patient education, early detection, and timely intervention can help preserve tooth structure and maintain oral function. Given the aging population, regular maintenance and long-term monitoring are critical to mitigating the functional and aesthetic consequences of DW.

## Data Availability

The original contributions presented in the study are included in the article/Supplementary Material, further inquiries can be directed to the corresponding author.
