# Supplementary material for: Prevalence and risk indicators of dental wear in a Saudi subpopulation
Source: Front Oral Health. 2025 Dec 8;6:1702554. doi: 10.3389/froh.2025.1702554 (PMC12719497; doi:10.3389/froh.2025.1702554)
Supplement: Supplementary file 1 [file Datasheet1.pdf]

TWI

**Age group:**	20-30 years	31-40 years	41-50 years	Above 50 years

**Gender**	Male	Female		
**Socioeonomical status**	High<br>Above 12000	Moderate<br>6100-12000	Low<br>6000 and below	
**Education**	None	Primary school	Secondary school	Bachelor or above
**Location**				

**1. Diet**	Beverages (Carbonated "Cola"-EnergyDrink-Fruit Juice)	Acidic Food (Orange, Grape Fruit, Lemon,Ketchup, Citrus Fruit)	Healthy diet
**2. Systemic condition**	Medically fit		Medically compromised

**3. Smoking**	Yes	No
**Pipe smoke**	Yes	No
**Cigarette smoke**	Yes	No
**Tobacco**	Yes	No

**4. Tooth brushing**	Yes	No		
**5. Type of toothbrush**	Soft	Medium	Hard	No use
**6. Brushing time**	Before meal	After meal	Both	No use
**7. Brushing tech**	Correct	Incorrect	No use	

**8. TMJ examination**	Normal	Clicking	Noise

**9. Masticatory muscle exam (Masseter, Temporalis)**			
Normal	Pain	Tenderness	Enlargement
